# Ureterolithiasis and the quest for rational use of diagnostic imaging methods

**DOI:** 10.1590/0100-3984.2018.51.6e2

**Published:** 2018

**Authors:** Jorge Elias Jr.

**Affiliations:** 1 Associate Professor in the Division of Imaging Sciences and Medical Physics, Head of the Department of Internal Medicine, Ribeirao Preto Medical School of University of Sao Paulo (FMRP-USP), Ribeirão Preto, SP, Brazil. E-mail: jejunior@fmrp.usp.br.. https://orcid.org/0000-0002-1158-1045.

Over the past 30 years, there has been an increase in the number of cases of urolithiasis at health care facilities in the United States, that number doubling between 1990 and 2010; consequently, urolithiasis now ranks among the ten most common complaints in emergency rooms^(^^1^^)^.That growth was five times greater than was that in the overall number of emergency room visits, in general, which shows the importance of this condition in the emergency setting^(^^1^^)^. Although there are differences among countries in terms of ethnicities, dietary habits, and climate, there seems to be a trend toward a global increase in the incidence of urolithiasis, with a proportional increase among females, although that incidence continues to be higher in white men between 45 and 64 years of age^(^^1^^)^.

The three major risk factors for urolithiasis in the general population are obesity, diabetes mellitus, and the use of dietary calcium supplementation^(^^1^^)^. Obesity has a direct negative impact on the accuracy of abdominal ultrasound, as has been shown in studies of conditions such as appendicitis^(^^2^^,^^3^^)^. Sauvain et al.^(^^3^^)^ found that ultrasound findings were inconclusive for the diagnosis of appendicitis in 42% of patients with a body mass index (BMI) ≥ 25 kg/m^2^, compared with 6% of those with a BMI < 25 kg/m^2^, suggesting that computed tomography should be the method of choice for patients who are overweight or obese. Keller et al.^(^^2^^)^ also demonstrated a high (49%) rate of nondiagnostic ultrasound examinations for appendicitis in overweight patients.

In an article published in the previous issue of Radiologia Brasileira, Nery et al.^(^^4^^)^ showed that the use of ultrasound can delay the diagnosis and treatment of suspected ureteral calculi for patients with a BMI > 27 kg/m^2^, because such patients will subsequently need to be evaluated by multidetector computed tomography (MDCT). The authors also demonstrated that for each unit increase in BMI there was a 16% increase in the rate of false-positive ultrasound results. Although there have long been indications that ultrasound has low accuracy in the abdominal evaluation of overweight individuals, the Nery et al. article^(^^4^^)^ is the first to provide such solid evidence regarding ultrasound evaluation in suspected cases of ureterolithiasis. This is a valuable information for the construction of clinical care protocols for this common clinical entity, allowing more rational allocation of resources and more rapid decision-making regarding the most appropriate treatment for such patients.

Obviously, decisions regarding the initial imaging method for the investigation of lumbar pain suspected to be caused by urolithiasis should take into consideration variables and parameters other than BMI, such as age, gender, the imaging methods available, and the expertise of the medical staff at the facility in question. One question that arises in this context is whether room exists for ultrasound as an exclusive method for the evaluation of ureteral calculi in symptomatic patients. In other words, is the information provided by a positive ultrasound result in the detection of the calculus sufficient? In that context, the fundamental findings are the position of the calculus, its size, and the presence or absence of other calculi. The position and size of the calculus have an influence on the immediate treatment, whereas the presence or absence of other calculi has an influence on decisions regarding systemic treatment for the prevention of additional calculi, given that urolithiasis recurs in only approximately 22% of clinically treated cases, compared with more than 90% of untreated cases. The indication for conservative clinical treatment, using drugs that facilitate the expulsion of ureteral stones, is still a controversial issue. However, after the publication of the first randomized studies, more than ten years ago, it has become relatively well established and is routinely used when the calculus is > 0.5 cm in diameter and there is no indication for emergency intervention, such as pyelonephritis, obstruction of a single kidney, and intractable pain^(^^5^^)^. It is of note that ultrasound can overestimate the size of a ureteral calculus, especially for calculi with a diameter ≤ 0.5 cm, although it is expected that such stones are likely to be eliminated spontaneously, without the need for pharmacological therapy^(^^5^^)^. It is also noteworthy that there have been refinements to the ultrasound technique that facilitate the evaluation of urolithiasis, such as the evaluation of the twinkling artifact^(^^6^^,^^7^^)^, ureteral jet examination using Doppler ultrasound^(^^8^^,^^9^^)^, and detection/characterization of hydronephrosis caused by a ureteral calculus^(^^10^^)^, findings that can have an impact on diagnostic accuracy and can facilitate case-by-case treatment. Therefore, there is the possibility that the performance of ultrasound, which is an innocuous and effective method in comparison with MDCT, will improve. Although MDCT is a more accurate method^(^^11^^)^, it carries the risks inherent to the use of ionizing radiation. To our knowledge, there have been no randomized studies comparing ultrasound and MDCT in the evaluation of ureterolithiasis. However, in 2014, Smith-Bindman et al.^(^^12^^)^ conducted a randomized multicenter study involving 2759 patients for nephrolithiasis evaluation. The authors showed that, for nephrolithiasis, there were no differences among the ultrasound performed by radiologists, the point-of-care ultrasound performed by the emergency room physician, and MDCT, in terms of high-risk diagnoses with complications, serious adverse events, pain scores, return emergency room visits, or hospitalization rates, the levels of radiation exposure being lower in the patients in whom the investigation began with ultrasound. A concern that should always be present is the unnecessary use of MDCT in general, but more specifically when the number of exams per patient is large, as can occur in cases of chronic pain and multiple episodes of ureteral stone passage^(^^13^^)^.

It is important to note that there is a validated clinical prediction system, known as the Sex, Timing, Origin, Nausea, Erythrocytes (STONE) score, that can identify patients with a high probability of uncomplicated ureteral calculi, who could benefit from the institution of treatment without undergoing MDCT^(^^14^^)^. However, the evaluation of patients on a case-by-case basis, considering the daily clinical routine, together with patient expectations and perceptions of the quality of medical care (including whether or not a medical test is requested), adds important variables to the equation of care for suspected cases of urolithiasis. In addition, the confidence provided by visualization of the calculus by the requesting physician, compared with the blind confidence in the outcome of an ultrasound examination, also affects individualized care in patient-centered medicine. In that sense, the study conducted by Nery et al.^(^^4^^)^ provides important information regarding current clinical practice; that is, what occurs in patients who undergo MDCT, which was used as a reference standard for determining the accuracy of ultrasound. It is of note that the ultrasound and MDCT results were both negative in 31.2% of the cases; that is, one third of the patients probably did not need to undergo MDCT after ultrasound. Therefore, when the ultrasound results are negative, the BMI is < 27 kg/m^2^, and there are no signs of hydronephrosis, clinical follow-up is probably the best course of action.

The abovementioned considerations indicate only the complexity of this theme, in which the consensus regarding the use of imaging methods in population-based health care has room to evolve. One major counterpoint is the fact that when we receive a request for ultrasound to investigate urolithiasis in a non-hospital setting, a situation that is very common, the examination should usually be performed following best practices, with the expectation that we will be able to resolve the case, regardless of the BMI of the patient in question. Although patient BMI has a direct effect on the diagnostic accuracy of abdominal ultrasound, it is not uncommon for patients with morbid obesity to have an ultrasound window that allows the diagnostic examination to be performed. In that context, it is important that the diagnostic impression be given after the examination and not as a prejudgment. That is certainly one of the factors that explains the great variability across studies evaluating the accuracy of ultrasound.

It should be borne in mind that, for the pediatric population and for pregnant women, ultrasound is the main method for the investigation of urolithiasis, being followed by magnetic resonance imaging if necessary^(^^15^^-^^17^^)^. Finally, when MDCT is indicated, there is evidence that strongly supports a recommendation for the use of a protocol with an up to 85% reduction in the radiation dose^(^^13^^,^^18^^)^.
